# Comment on “The Impact of Chronic Tobacco Smoking on Retinal and Choroidal Thickness in Greek Population”

**DOI:** 10.1155/2016/1958086

**Published:** 2016-06-28

**Authors:** Salih Uzun

**Affiliations:** Etimesgut Military Hospital, Department of Ophthalmology, 06790 Ankara, Turkey

We have read the article entitled “The Impact of Chronic Tobacco Smoking on Retinal and Choroidal Thickness in Greek Population” by Moschos et al. [1] with interest. We congratulate the authors for demonstrating that the choroidal thickness (CT) and ganglion cell complex (GCC) thickness were significantly reduced in smokers when compared to the control group. We would like to ask for further details and contribute to the article.

Advancements particularly in optical coherence tomography (OCT) technology enabled high-resolution and noninvasive imaging of the retina and choroid. A number of local and physiological/pathological conditions may affect CT [2]. On the other hand, CT shows a significant diurnal variation. It increases at night and gets thinner later in the day. Tan et al. demonstrated the highest mean CT as 372.2 *μ*m, which occurred at 9 AM [3]. The mean CT then decreased progressively over the subsequent time points to a low of 340.6 *μ*m at 5 PM.

Usui et al. studied subfoveal choroidal thickness (SFCT) in healthy subjects and measured SFCT every 3 hours over a 24-hour period [4]. They found that the mean SFCT was the thinnest (271.9 ± 103.5 *μ*m) at 6 PM, and it was the thickest (290.8 ± 110.8 *μ*m) at 3 AM. In that study, Usui et al. showed that diurnal variation of CT might be up to 65 *μ*m (range, 8–65 *μ*m).

However, in their study, Moschos et al. did not mention the time at which OCT was obtained. Therefore, one might expect that physiological fluctuation would have significantly affected the test results and hence the statistical analysis. We recommend performing OCT measurements at the same time point, every day.
